# Borrowing the Features of Biopolymers for Emerging Wound Healing Dressings: A Review

**DOI:** 10.3390/ijms23158778

**Published:** 2022-08-07

**Authors:** Ioannis Gardikiotis, Florina-Daniela Cojocaru, Cosmin-Teodor Mihai, Vera Balan, Gianina Dodi

**Affiliations:** 1 Advanced Research and Development Center for Experimental Medicine (CEMEX), Grigore T. Popa University of Medicine and Pharmacy of Iasi, 9-13 Kogalniceanu Street, 700454 Iasi, Romania; 2 Biomedical Sciences Department, Faculty of Medical Bioengineering, Grigore T. Popa University of Medicine and Pharmacy of Iasi, 9-13 Kogalniceanu Street, 700454 Iasi, Romania

**Keywords:** wound dressing, phases of healing, biopolymers, platelet-rich plasma (PRP), growth factors, stem cells

## Abstract

Wound dressing design is a dynamic and rapidly growing field of the medical wound-care market worldwide. Advances in technology have resulted in the development of a wide range of wound dressings that treat different types of wounds by targeting the four phases of healing. The ideal wound dressing should perform rapid healing; preserve the body’s water content; be oxygen permeable, non-adherent on the wound and hypoallergenic; and provide a barrier against external contaminants—at a reasonable cost and with minimal inconvenience to the patient. Therefore, choosing the best dressing should be based on what the wound needs and what the dressing does to achieve complete regeneration and restoration of the skin’s structure and function. Biopolymers, such as alginate (ALG), chitosan (Cs), collagen (Col), hyaluronic acid (HA) and silk fibroin (SF), are extensively used in wound management due to their biocompatibility, biodegradability and similarity to macromolecules recognized by the human body. However, most of the formulations based on biopolymers still show various issues; thus, strategies to combine them with molecular biology approaches represent the future of wound healing. Therefore, this article provides an overview of biopolymers’ roles in wound physiology as a perspective on the development of a new generation of enhanced, naturally inspired, smart wound dressings based on blood products, stem cells and growth factors.

## 1. Introduction

Wounds are defined as the derived impairment of biological tissue integrity, including skin, mucous membranes and organ tissues, caused by various types of trauma or as a result of any underlying medical or physiological condition [1]. The World Health Organization classifies wounds in its management guidelines [2] as: (i) clean; (ii) clean-contaminated; (iii) contaminated and (iv) dirty-infected, which have been further labelled as four classes by Herman and Bordoni [1], with an organization based on the microbial/external contaminant burden. However, this classification is not applicable for all types of wounds or healthcare providers; therefore, other classification schemes are also available, since a proper taxonomy helps physicians to predict potential infections, complications and re-interventions. Several other arrangements, such as the Gustilo–Anderson classification, the Tscherne classification, the Arbeitsgemeinschaft Osteosynthesefragen soft tissue grading system and the Red Cross wound classification [3], are also used to describe wounds and guide their management. Overall, wounds can be classified based on the type of repair process involved (acute or chronic), aetiology (superficial, incised, crush, lacerated, stab, contused or secondary), type (simple or combined), depth (superficial, partial or full thickness) and clinical appearance (fresh or old) [4,5]. This variety has also resulted in a wide range of wound dressing types.

Innovations in the variety of wound dressings, frequently introduced to target different aspects of the wound care process, have improved patients’ lives over the years through enhancements in the accessibility and flexibility of treatments.

Dressings are classified based on various criteria, briefly described below:

- Function in the wound: debridement, antibacterial, occlusive, absorbent, adherence;
- Type of material: hydrocolloid, alginate, collagen;
- Physical form: ointment, film/membranes, foam, gel, spray, composite, particulate systems;
- Type of physical contact with the wound: primary, secondary or island dressings;
- Traditional biomaterial based-dressings or artificial dressings;
- Mechanism of action: passive (protects the wound area with no direct effect on the wound) or interactive (produces an optimum environment at the wound dressing interface).

Broussard and Powers [6] developed a wound assessment and dressing selection flow chart in order to match these types to different wounds, and healing phases, since the dressing type may change as the wound heals. Thus, before selecting a dressing, several factors, such as the underlying cause of tissue damage, tissue perfusion and bacterial load, should be considered. The classical dressing protects wounds from bleeding, dehydration, deposition of microorganisms and physical and mechanical damage.

The features of an ideal wound dressing from the specialist point of view, as summarized by Broussard and Powers in 2013 and refined in 2014 [7] and, more recently, in 2020 [8], are briefly listed below:

- Suitable design, with minimal inconvenience to the patient (easy to apply, maintain and store; adaptable to the wound shape; proper elasticity; and high mechanical strength);
- Removes and absorb excessive exudate drainage from the wound;
- Easily sterilized;
- Cost-permissive;
- Induces rapid healing;
- Preserves the body water content and temperature of approximately 37 °C;
- Gas-permeable;
- Ability to release bioactive ingredients when connecting with the wound surface;
- Non-adherent and non-allergenic, biocompatible;
- Easy removal at the end of treatment and/or biodegradable;
- Provides barrier to external contaminants;
- Stimulates growth factors and production of granulation tissue and re-epithelialization;
- Minimizes trauma or maceration to wound edges.

However, it is well-established that different people see wound treatment differently, and only the patient understands the significance of a proper healing process; therefore, the ideal wound dressing should combine various features. Over the years, several studies have aimed to achieve more effective wound therapies that would be able to re-establish the damaged cells, provide scar-free healing, reduce health costs and offer long-term relief. For this reason, conventional dressings may be susceptible to upgrades regarding the encountered drawbacks mentioned above in terms of aesthetic and possible functional alterations. In this review paper, we briefly introduce the main aspects of wound physiology along with the wound healing properties of selected naturally derived polymers, with a particular focus on the restoration events involved. Lastly, we highlight the growing interest in the use of biopolymer features borrowed from nature for the development of a new generation of smart wound dressings based on emerging therapies.

## 2. Overview of Current Status

Numerous review papers in the literature deal with wound healing settings and describe in detail their different aspects, including the main phases, the common wound healing dressings, their key advantages and limitations and the need for dressings with enhanced characteristics.

A review worth mentioning from 2007 [5] presents, besides the wound healing physiology and the factors that impair wound healing, a method for effective wound management to ensure successful practice. Several desirable functions need to be considered in the choice of wound dressing: debridement—to remove necrotic tissue or foreign materials; hydration and adsorption preservation; gaseous exchange; infection prevention; normal temperature provision; non-adherence; and cost-effectiveness. All these key management points are still pertinent, and they are addressed by the various types of dressings, which can be classified as primary, secondary and island dressings, as described in 2006 by van Rijswijk [9], or as traditional, modern and advanced dressings; skin replacement products; and wound healing devices. It is important to mention that polymeric dressings, which include ALG, Cs, pectin and HA, are considered the most modern dressings directly applied to the surfaces of wounds, and they are the most widely studied. After more than 10 years since their development, there are still many unexplored polymeric dressings with specific properties that are required for effective healing processes.

In 2010, the important issue of the psychological stress and pain that can delay wound healing was explored by Solowiej et al. [10]. The authors discuss the clinicians’ management approach, which should include selection of appropriate dressings that minimize patient distress and pain during the wound care process. Specific characteristics—namely, good adherence to skin, permeability and absorption—should be the focus of consideration when selecting a dressing in order to reduce patient pain and discomfort. Acrylates, polyurethanes, hydrocolloids, soft silicone and non-adhesive alternatives, such as pastes and non-adherent foams are several atraumatic dressings identified by the World Union of Wound Healing Societies that significantly reduce levels of pain at dressing change.

In 2015, Landriscina et al. [11] provided a systematic tool for dermatologists to better assess and treat chronic wounds, covering the basic and necessary aspects of the types of available dressings then available. The wound management of the 21st century was upgraded by Lindholm and Searle’s [12] review paper one year later, which described wounds as “The Silent Epidemic”. The authors pointed out that the costs of three factors—reducing healing time, optimising dressing change frequency and preventing complications—can be substantially reduced, since 80–85% of the total costs in wound management are represented by nursing time and hospital costs and not the wound dressings per se.

More recently, review papers found on specific platforms from the last three years point out several key points regarding the selection of appropriate wound dressings for various wounds. The paper by Ghomi et al. [13] presents, for the first time, a detailed classification of wound dressings based on their origin—namely, animal, herbal and synthetic dressings—along with the characteristics of the ideal wound dressing and adequate requirements for the selection of dressings. The authors state that there is still no suitable product for chronic wounds, such as diabetic wounds and venous leg ulcers, among the 3000 dressings identified on the market; therefore, further studies are crucial to find enhanced treatment options for wound healing.

Shi et al. [8] described the history of the development of wound dressing and of the classification of modern wound dressings, remarking that there are no dressings that can achieve all required functions. The manuscript was presented as a clinical guideline for the selection of suitable wound dressings for effective wound healing based on particular conditions, such as the patient’s primary disease, the features of the dressing and, specifically, the physiological mechanisms of wounds.

Emerging technologies for skin wound care and regeneration based on key components of regenerative medicine, such as growth factors, autologous cells and stem cells, gene therapy and tissue engineering, were introduced by Tottoli et al. [14] in 2020. Despite the enormous advances in wound healing therapies, the authors point out that skin regeneration is a challenge that requires close collaboration between researchers in many disciplines. One interesting perspective from this research group refers to three-dimensional bioprinting combined with electrospinning, using bioinks suitable for different types of cells on one side and biodegradable and biocompatible polymers on the other side. However, can this initiative overcome the cell damage caused by the 3D bioprinting process or the poor mechanical resistance of scaffolds?

The prospects for deeper collaboration between multiple disciplines were also investigated by another review paper in 2021 by Hawthorne et al. [15], who pleaded for interplay between the medical and scientific research fields, which is vital for translating new discoveries from the lab to treatments at the bedside. After a detailed analysis of the dressing types, biomaterials and disciplines pertinent to wound dressing developers, the authors concluded that further exploration of the use of biomaterials in the synthesis of hydrogels, utilizing electrospinning and incorporating a drug delivery system and nanoparticles in the matrix of the wound dressing, could result in innovations in wound dressing technology.

A paper by Mirhaj et al. [16] critically reviews the emerging treatment strategies in wound care currently under development, as well as those used in clinical practice. As underlined in the review, there are many available therapies but none are 100% effective; therefore, the treatment of wounds is still considered an unmet clinical need. In the same context, the authors consider that an interdisciplinary approach, including clinicians, material and biological scientists and engineers, is necessary to develop effective therapies for chronic wounds in the near future, which could be based on 3D printing, combinations of new smart biomaterials, antibacterial drugs and nanoparticles with stem/adipose cells and/or blood products.

We can analyse the feasibility of the proposed perspectives, by comparing conventional dressings with the innovative trends in regenerative medicine. Therefore, Table 1 provides a comparative analysis of commercial dressings, biopolymers and molecular biology perspectives based on the available research data for each generation. Different examples of commercial dressings can be found in the table and, for the other products, a considerable number of models are described in Section 5 and in the two tables of Section 6 of the paper.

Overall, to the best of our knowledge after analysing most of the available review papers from the last 5 years, along with the features of the presented dressings, there are currently no reviews of biopolymer-based wound dressings that discuss them as the starting base for emerging therapies based on molecular biology.

## 3. Methods

A comprehensive literature search was performed in the PubMed.gov, Science Direct, Web of Science and Scopus databases using the following keywords: “wound”, “healing”, “dressing”, “biopolymers”, “regenerative medicine”, “platelet rich plasma”, “growth factors” and “cold plasma”. For example, the literature search in PubMed.gov using the terms “wound”, “healing” and “dressing” returned nearly 2135 reviews from across all years, of which more than 365 were published in the last two years (2021 and 2022). Each section of our review includes the relevant review papers obtained with the above-mentioned keywords from the period 2017–2022. Moreover, in each section, we provide the analysed period for the specific research articles. We are confident that using the different combinations of keywords made it possible to obtain the relevant previous studies in the field, with special attention given to emerging therapies and their wound-healing efficacy. The goal was to provide a comprehensive overview of the potential of and the benefits expected from current protocols and innovative combinations of modern and advanced dressings.

## 4. Wound-Healing Cascade

The term “wound” is defined as the disruption of the anatomical structure and function of the skin, as it is the most exposed to tissue damage, but also of subcutaneous tissue, muscles, bone, tendons, nerves and vessels [24]. As the lesion appears, the body begins the restoration of the physiological conditions through a wound-healing cascade of regulated, interconnected events to achieve wound healing [25]. The four phases of the entire wound-healing process—namely, haemostasis, inflammation, proliferation (granulation, vascularization, wound closure) and remodelling—overlap with one another and require at least one year in total for a long-lasting result. It is important to specify that these stages are not separate but form a continuum in the wound healing process based on specific events [26].

The wound healing phases involved in this dynamic and complex process have been extensively described in many books and reviews over the years, in which more details can be found [27,28]; therefore, we intend to briefly present a compressed overview for the reader.

The first “visit” in the wound area is made during the haemostasis phase, which assures blood coagulation through intrinsic and extrinsic pathways and the formation of a blood clot comprising platelets and fibrin. The lifesaving vascular response involves the appearance of the blood-clotting cascade; namely, factor FXII, high-molecular-weight kininogen, platelet factor 3, red blood cells, tissue factor, tissue factor pathway inhibitor, thrombomodulin and white blood cells. The formed clot serves, besides the obvious role preventing blood loss, as a first line of defence against microbial invasion and an interim base for the homing of blood-borne cells, thanks to regulatory function of cytokines and growth factors. The formed fibrin crosslinks, entraps platelets and adheres to the matrix through integrins. Fibrin binds to integrin CD11b/CD18, fibroblast growth factor-2 (FGF-2) and vascular endothelial growth factor (VEGF) for wound tissue vascularization and to insulin-like growth factor-I for cell proliferation. The thrombin foundation represents the first sign for the next “visit” to the wound site; namely, the cellular response. The thrombin also activates the inflammatory process by releasing IL-1α, IL-1β, IL-6 and TNF-α [29]. The entrapped platelets stimulate the release of FGF-2, IGF-1 and TGF-β for collagen synthesis, activate the transformation of fibroblasts to myofibroblasts (TGF-β), initiate angiogenesis (FGF-2, VEGF-A, HIF-1α, TGF-β) [30] and support re-epithelialization activity due to the presence of EGF, FGF-2, IGF-1 and TGF-β [31].

The inflammation phase, triggered by the release of inflammatory cytokines in the previous phase, represents the response of the wound site in preparation for the wound closure. This step involves the recruitment of neutrophils and monocytes, the differentiation of macrophages and lymphocyte infiltration to the site of the skin injury and occurs over the following 2–5 days. The presence of neutrophils is crucial due to their capacities for phagocytosis, infection control and cleansing of devitalized tissue, but also due to their activity as chemo-attractants for other cells involved in this phase (cytokines, proteases and growth factors). The activated pro-inflammatory cytokines, such as IL-1β, TNF-α and interferon-γ, induce the expression of adhesion molecules, such as P-selectin and E-selectin, which bind with integrins expressed on the cell surface of neutrophils. Furthermore, IL-8, MCP-1 chemokines and growth-related oncogene-α interact in addressing acute-phase inflammation after injury. Other products dispensed by neutrophils to the wound site include antimicrobials, such as cationic peptides and proteases, which are important for wound healing progression [32], and neutrophil extracellular traps (NETs), which respond to pathogens such as bacteria, fungi and viruses [33]. The existing macrophages “take care” of the dead cells and debris and, in addition, they deliver a wide range of growth factors, such as TGF-β, TGF-α, basic FGF (bFGF), VEGF and PDGF, that enable wound healing through cell proliferation, synthesis of extracellular matrix (ECM) and angiogenesis [34].

The proliferative phase overlaps with the inflammatory period and mediates re-epithelialization, the formation of new blood vessels, influx of fibroblasts and establishment of the ECM. During the 3 week period, the endothelial cells invade the wound and fibroblasts seal that area. Migrating and activated fibroblasts produce cytokines for keratinocyte migration and proliferation, as well as PDGF and TNF-α, which also produce keratinocyte growth factor, responsible for wound re-epithelialization [35]. During this phase, the granulation tissue initially consists of a network of type III collagen, and it is, later replaced by type I collagen, existing in scar tissue.

Wound vascularization, achieved through angiogenesis or vasculogenesis, contributes to all wound healing phases, including the haemostatic plug, which provides a suitable environment for blood-borne cells, platelets, which function as a source of growth factors, and cytokines, which recruit endothelial cells to the wound site. Then, the leukocytes from the inflammatory phase lay the foundation for wound tissue vascularization with the aid of VEGF-A and IL-8, these being major sources of pro-angiogenic factors. Furthermore, the macrophage-derived TGF-β, TGF-α, bFGF, PDGF and VEGF determine the skin wound angiogenesis in this phase. Therefore, after the production and release processes, the angiogenic factors bind to the endothelial cell receptors, which activate, proliferate and migrate the microvascular endothelial cells for directional movement. A critical event in the formation of new vessels is the ECM remodelling, mainly since it controls cell growth, migration and differentiation during different stages of angiogenesis [36]. The angiogenesis process continues with the formation of capillary-like tubes specific to endothelial cells, which shield on a reconstituted matrix, attach, align and shape the vessels [37]. The next event is loop formation and pericyte coverage for vascular stabilization, a process managed by VEGF and PDGF [38]. However, the pericyte functions are not only involved in angiogenesis but also in wound healing, since the skin pericytes represent a powerful stem cell population capable of altering the ECM microenvironment and promoting epidermal tissue renewal from non-stem cells [39].

The last phase of the wound healing process is wound remodelling, which contributes to the closure of wounds by the fibroblasts. The restoration of the epidermal barrier is based on the re-epithelialization process and results from three overlapping keratinocyte functions: migration, proliferation and differentiation [40]. These events, through which keratinocytes accomplish the task of re-epithelialization, are enhanced by a moist setting and facilitated by the enzyme matrix metalloproteinase 1, a collagenase that reduces the affinity of collagen–integrin contacts [41]. The re-epithelialization continues until the injured tissue is restored and the wound gradually becomes less vascularized.

## 5. Biopolymers as Tuneable Materials

**Figure 1:**
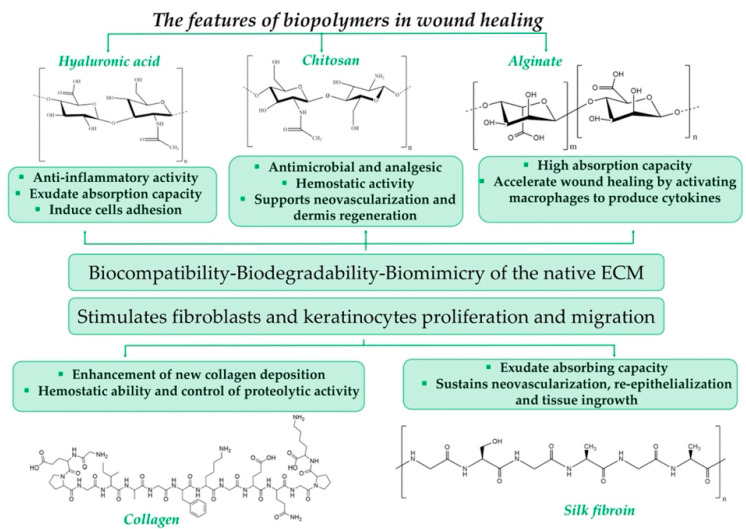
The features of biopolymers in wound healing.

Biopolymers, in the form of polysaccharides or proteins, have attracted the interest of the scientific wound community due to their impressive features, such as excellent biocompatibility, biodegradability, bioactivity and bioresorptivity; non-toxicity; rapid manufacturing; and, most important, the highest level of biomimicry of the native ECM [42]. Figure 1 highlights the features of some of the most commonly used biopolymers in wound healing.

Even if several disadvantages can also be highlighted—namely, batch-to-batch dissimilarities, limited shelf life and their mechanical properties—researchers have managed, over the years, to obtain complex biopolymer-based architectures through appropriate processing [43]. Therefore, the obtained tuneable structure, comparable with the native ECM, has turned out to be suitable for dressings applicable in clinical practice [44].

By analysing the ClinicalTrial.gov database, we identified numerous biopolymeric dressings/devices that have been exploited in clinical trials in recent decades, as presented in Table 2. Several biomaterials with appropriate biological properties to serve as wound dressings can be identified; namely, chitosan, alginate, silk fibroin, collagen, cellulose and hyaluronic acid.

However, let us go back and explore the great quantity of available data that validated the above-mentioned natural polymers for the fabrication of commercially available wound dressings. From the 133 accessible review papers found on the PubMed platform with “biopolymer wound healing” as the keywords, we highlight only a few of them, especially those from the last two years, in order to underline the perspective of this paper.

A recent review paper by Yang et al. [46] introduces the polysaccharides with good biological properties—namely, alginate, cellulose, chitosan, hyaluronic acid and their derivatives—used to prepare self-healing hydrogels, along with their preparation methods and potential applications in biomedicine. The paper highlights how polysaccharide features make them a good choice to improve the biocompatibility, biodegradability and cell support of hydrogels. The relevance of biopolymeric nanosystems based on chitin, chitosan, collagen, alginate, fucoidan, gelatine, silk fibroin and hyaluronic acid as potential wound-healing structures are discussed in the review by Moholkar et al. [47] from 2021. The authors dedicate a section to clinical applications and challenges where they briefly mention that chitin and chitosan wound dressings are already available on the market, and fucoidan-, alginate-, silk fibroin- and hyaluronic acid-based wound dressings have limited applications in the clinical trials due to lack of knowledge and research in the field. From the same year, Gobi et al. [48] provide an overview of biopolymers and synthetic polymer-based nanocomposites with wound dressing applications. The authors focus on metal/metal oxide nanomaterials, such as zinc oxide, cerium oxide, silver, titanium oxide, iron oxide and other materials (graphene and carbon nanotubes), embedded in biopolymeric matrices due to their antimicrobial properties, which make them suitable for use in wound healing. Nosrati et al. [49], in their review paper, discuss the structure and properties of carbohydrate polymers, physical and chemical crosslinking of polysaccharides, scaffold fabrication techniques and the use of polysaccharide-based scaffolds in wound-healing applications. The review paper by Raina et al. [50] from 2020 covers, besides the potential biopolymers used for treating chronic wounds and their capabilities in the development of innovative systems for drug delivery, the recent patents and clinical trials for nanotechnology-based polymeric formulations for wound healing.

Homaeigohar and Boccaccini’s [51] review paper details the developments in antibacterial biopolymeric nanofibrous wound dressings from 2015 to 2020 based on a comprehensive literature analysis. The authors give particular attention to those made of bio-hybrids, containing antibacterial nanoparticles, nature-derived compounds and biofunctional agents, which have been observed to promote healing rates. The authors have an interesting point of view regarding various aspects related to chemistry and composition, synthesis, design and engineering, multifunctionality and testing approaches, aspects that nanofibrous wound dressings must pay attention to.

A similar point of view was also embraced in this study when analysing the available review papers related to biopolymer dressings from the last 5 years; specifically, that, at present and to the best of our knowledge, this is the first study that discusses biopolymer-based wound dressing as the starting base for emerging therapies based on molecular biology. In this context, the following sections focus on the applications of the most widely used biopolymers in wound healing, discussing for each polymer the established stage of the healing cascade that is addressed.

### 5.1. Chitosan (Cs)

Cs, one of the most promising biopolymers for wound healing, is rarely found in nature, being obtained by deacetylation of chitin [52,53]. Desirable characteristics, such as biodegradability, non-toxicity, low price and biocompatibility, recommend it for regenerative medicine applications, since it facilitates wound closure, supports neovascularization and regeneration of dermis and shows antimicrobial, analgesic and haemostatic activities [53,54]. Due to its rigid crystalline structure and insolubility in water, derivatives of Cs are prepared through chemical modifications that introduce hydrophilic groups to their structure, a move that addresses the identified drawbacks. The most commonly studied water-soluble Cs derivatives are O-carboxymethyl Cs, N-(2-carboxyethyl)-Cs and N-succinyl Cs (NSC) [54].

In the United States, the FDA recognizes Cs as safe for tissue-engineering and wound-dressing applications, and in other countries, such as Finland, Italy and Japan, it is included in the dietary supplement category. As Matica et al. summarize in a comprehensive review from 2019, Cs is used in 24 wound dressings available on the market [55].

According to the existing data summarized by Feng et al. [56] in their latest review, chitosan and its derivatives are active mainly in the first three stages of wound healing:

- in haemostasis—by stimulating the aggregation of platelets and erythrocytes and restraining the dissolution of fibrin;
- in inflammation—through its well-known and validated antibacterial properties;
- in proliferation—by promoting the growth of macrophages, fibroblasts and capillaries.

A brief search in the Pubmed.gov platform using the keywords “haemostasis”, “wound dressings” and “chitosan” resulted in more than 700 research articles and reviews. Considering this number, in the following paragraphs we only summarize data from several representative studies, which highlight the properties that make wound dressings based on Cs suitable for wound healing, different animal models and manufacturing techniques.

Recently, Biranje et al. [57] combined Cs with carrageenan, another, oppositely charged, polysaccharide, and obtained non-cytotoxic composite dressings with excellent in vitro haemostatic properties demonstrated in a human thrombin-antithrombin assay.

Cs mixed with different combinations of chloride, aluminium sulphate hydrate or iron (III) sulphate and levofloxacin, manufactured as wound dressings, was proved to have encouraging haemostatic competence after evaluation of whole blood clotting time, plasma recalcification time, platelet adhesion and in vivo tests in mice [58].

Hydrogels based on Cs loaded with β-cyclodextrin polyester (CDPE) were analysed in acute liver punch models of pigs, rabbits and rats and showed promising results due to superior their haemostatic properties compared to commercial dressings [59]. In another study, hydrophobically modified Cs combined with α-cyclodextrin, an amphiphilic supramolecule, also showed efficient in vitro haemostasis in rheology studies and also in vivo, reducing the bleeding time in a rat model by 90% [60].

Another mix based on Cs with haemostatic properties proved in vivo in two rat models (tail amputation and liver laceration) was prepared and analysed by Sun et al. [61]. The authors used an inverse emulsion method and thermally induced phase separation for the fabrication of porous Cs microspheres mixed with kaolin, a mineral clay [61]. Moreover, in 2015, the US military frequently used kaolin-impregnated gauze as primary haemostatic dressings. However, in time, kaolin-impregnated gauzes were replaced or used complementarily with several dressings based on Cs that are part of the third generation of dressings [62].

Using a facile radical polymerization process, Cs was grafted with methacrylate, N-hydroxymethyl acrylamide and dopamine to obtain a bionic hydrogel with excellent adhesion properties. The hydrogel showed excellent haemostatic properties after evaluation in mouse liver haemorrhage models and, more complexly, in beating rat and rabbit hearts. The adhesion properties of the hydrogel significantly stopped the bleeding of the beating heart, which, even after 5 min under dynamic blood pressure flow, remained at the fixed site [63].

The water-soluble Cs derivate N-succinyl chitosan (NSC) showed good adhesion and humidity-retaining ability. The potential for the use of NSC in skin wound healing was evaluated in rabbits with full-thickness skin wounds created on the dorsum. The results of the in vivo studies proved that the NSC powder had antibacterial properties, supporting epithelialization, granulation and tissue development [54]. Furthermore, hydrogels based on Cs and honey demonstrated antimicrobial and healing properties in a rat full-thickness wound model [64], while antibacterial electroactive self-healing hydrogels obtained by mixing quaternized Cs-g-polyaniline with a complex synthetic co-polymer showed blood clotting properties and a capacity to sustain tissue development that were superior to a commercial hydrogel in a female Kunming mice full-thickness wound model [65].

In a rabbit femoral injury test, N-alkylated chitosan/graphene oxide sponges stopped excessive bleeding. A commercial Cs-based haemostat powder and standard medical gauze were tested in the same conditions and the Cs sponges were demonstrated to be easier to handle. Furthermore, the sponges were proved to have other characteristics that recommended them as wound dressing materials; namely, haemocompatibility, biocompatibility, mechanical stability and absorption capacity [66].

Another efficient, simple and also low cost manufacturing technique was described by Zheng et al. [67]. The authors obtained a haemostatic material by coating carboxymethyl Cs, gelatine and alginate on cotton gauze layer-by-layer that demonstrated a strong fluid absorption ability and good haemocompatibility and biocompatibility. In order to study the in vivo haemostatic performance of the composite dressing in comparison with cotton gauze, two animal models were chosen: a mice-tail amputation model and a mice liver injury model. The dressing proposed by the authors showed superior haemostatic properties [67].

Chemical crosslinking is often used in the manufacturing of tissue engineering products, including wound dressings, to improve their physical and biological features. The functional properties of some polysaccharides, such as Cs, xanthan, cellulose and starch, can be enhanced using sodium trimetaphosphate, a safe and non-toxic crosslinking agent [68]. Chen et al. [69] described a prepared porous starch/chitosan composite that was efficiently crosslinked with sodium trimetaphosphate. An in vitro blood cell evaluation experiment exhibited a synergistic haemostatic effect. Furthermore, the composites’ in vivo haemostatic performance in tail amputation and liver laceration rat models were shown to be adequate for the proposed application [69].

Three-dimensional (3D) printing is widely used in the fabrication of scaffolds for tissue engineering, films and membranes for wound healing, artificial tissues and even artificial organs [70,71]. The main advantages of the scaffolds prepared with this technique are their superior flexibility, controlled porosity and reproducibility [70]. For example, Hu et al. [71] prepared a supramolecular hydrogel using dual crosslinking from a complex mixture of PEGylated Cs, α-cyclodextrin (α-CD) and gelatine. The obtained bio-ink had excellent biocompatibility and, very importantly, tuneable strength.

Park et al. [72] prepared a hydrogel based on catechol-conjugated glycol Cs and evaluated it in comparison with the versatile Cs-catechol hydrogel, which is often studied as a haemostatic material, nanocomposite and 3D printing ink. The two types of Cs hydrogels were tested both in vitro, to study their cellular toxicity, and in vivo, to study skin irritation, immunoreactivity after subcutaneous implantation of hydrogels into mice and haemostatic properties, using a liver bleeding rat model. Glycol Cs-catechol showed better in vitro cytocompatibility and essentially improved the reported immune response to Cs-catechol, preventing uncontrolled immune protein adsorption and decreasing foreign reactions. The ethylene glycol groups did not considerably modify the adhesiveness or haemostatic ability of the hydrogels [72].

The manufacturing of hydrogels as beads gained serious consideration in many biomedical fields, such as drug delivery, protein release and absorption, due to their versatile form. Based on their macromolecular hydrogen-bonded network, Cs beads are usually obtained by precipitating the biopolymer in alkalis, dissolving it in acids or using crosslinking processes. Crosslinking may sometimes be tedious and crosslinkers may affect the biocompatibility of Cs biomaterials [73]. To avoid these drawbacks, a facile flow injection method for the preparation of Cs beads has been proposed by Li et al. [73]. A solution obtained by dissolving high-molecular-weight Cs into 1-ethyl-3-methylimidazolium acetate ionic liquid was flow-injected into ethanol, resulting in uniformly sized Cs beads. The beads showed excellent features, including swelling capacity, in vitro biocompatibility and haemostatic properties [73].

Cs is often combined with other biopolymers and processed in a variety of formulations in order to obtain dressings with improved properties that can induce haemostasis and support wound healing. Microparticles based on two of the most widely used biopolymers, carboxymethyl Cs and HA, were mixed with other two natural materials, starch and tannic acid, as crosslinkers, resulting in antibacterial 3D network structures with haemostatic properties that could repair a wound in 14 days [74]. In other studies, Cs and HA were used as the components of haemostatic composite sponges, incorporating fibrin nanoparticles loaded with growth factors [75] or tranexamic acid [76]. Haemostatic films are another form in which various combinations of biopolymers are often processed for wound-healing applications, such as those reported by Wu et al., which combined HA, CS and type I collagen [77].

Cs and ALG have also been successfully combined in a haemostatic dressing. Sponges based on carboxymethyl Cs, ALG and a Chinese medicine were made by He et al. [78] via green crosslinking and were proved to induce rapid haemostasis and sustained wound closure in a rat full-thickness wound model. Multi-resorbable haemostatic dressings composed of Cs, sodium/calcium ALG and/or carboxymethyl cellulose were prepared with lyophilisation and spray-drying techniques [79].

As anticipated, chitosan and its derivatives exhibit excellent haemostatic properties but, as mentioned above, they also promote anti-inflammation and proliferation of granulation in wound repair. Quercetin was incorporated into Cs nanoparticles with the aim of improving one of the drug’s limits: poor skin penetration. These nanoformulations sustained wound healing in Wistar rats through regulated modulations of pro-inflammatory cytokines and growth factors, even at low drug concentrations [80]. In another study, Cs and a metal ion-based hydrogel were incorporated into medical gauzes. In vivo studies on male C57BL/6 mice revealed that the gauzes loaded with Cs/metal ions promoted wound healing in different ways, including constraining inflammation by regulating the expression of inflammatory factors and building up granulation and through collagen deposition and maturation and angiogenesis, and they also showed antibacterial properties in infected tissues [81].

Three water-soluble Cs derivatives, N-carboxymethyl Cs, 5-methyl pyrrolidinone Cs and N-succinyl Cs, were processed separately as wound dressing foams and studied in parallel. The 5-methyl pyrrolidinone Cs foam showed the most appropriate behaviour in contact with fluids (water-vapour and water-swelling properties), the best drug delivery profile and non-cytotoxic in vitro activity. This Cs foam was loaded with a neuropeptide named neurotensin that acts as an inflammatory modulator in wound healing, and it was applied to control/diabetic mice wounds to determine its abilities. In the first days after implantation, the dressing induced fast healing in the early phases of wound healing in diabetic mice, decreasing inflammatory cytokine expression and reducing the amount of inflammatory infiltrate. Lately, after ten days, fibroblast migration and collagen expression and deposition increased considerably, demonstrating the role of chitosan in all the phases [82].

### 5.2. Hyaluronic Acid (HA)

Together with collagen, HA was one of the first biopolymers studied for wound healing, as observed after a brief search on the PubMed.gov database with “hyaluronic acid” and “wound healing” as keywords. The first studies appeared to be from 1952 and, since then, almost 2000 articles have been published, of which 240 are review papers.

HA, a remarkable non-sulphated glycosaminoglycan (GAG) with a large molecular weight but a simple structure is the main component of skin extracellular matrix(ECM), and it has a valuable role in inflammatory, angiogenesis and tissue regeneration processes. The features of HA are strongly influenced by its molecular weight: low-molecular-weight HA acts as a proinflammatory molecule, while high-molecular-weight HA acts as an anti-inflammatory and immunosuppressive molecule [83,84]. The structure of HA confers it a highly hydrophilic character, one of its main features recommending it for wound-dressing applications, making it useful for the absorption of exudates and for cell adhesion. Furthermore, it is well-known that this GAG has excellent biocompatibility and adequate biodegradability. In wound healing, it is used as a dressing and is manufactured in many forms—hydrogels, films, sponges, electrospun membranes and so on—depending on its application: for haemostasis, inflammation phases or to sustain proliferation and re-epithelialization processes [83,85].

A hybrid 3D microporous hydrogel was obtained by combining aminoethyl methacrylate HA and methacrylate methoxy polyethylene glycol and then loading the product with chorhexidine diacetate nanogels. The drug was gradually released over a period of 240 h, which ensured antibacterial activity for 10 days. In vivo, in a mouse model, the hybrid hydrogel showed the capacity for rapid haemostasis and increased wound healing [86].

The HA-pullulan film fabricated by Haiying et al., which also has the capacity for rapid haemostasis, evidenced excellent swelling abilities, meaning that it can be considered as a potential dressing to protect the wound bed from the accumulation of exudates and reduce the frequency of replacement [87]. Biodegradable nanofibrous scaffolds based on polycaprolactone, HA and encapsulated epidermal growth factor were synthesized using an emulsion electrospinning technique. In vitro, HA and the growth factors sustained human skin keratinocyte and fibroblast proliferation and infiltration and up-regulation of the wound healing-related genes collagen I, collagen III and TGF-β. Further, in a full-thickness wound model, the scaffold also showed a haemostatic ability [88].

In order to shorten the inflammatory stage and sustain angiogenesis using a gene complex, Yang et al. [89] developed a complex hydrogel with a hierarchical micro/macro-structure, crosslinked via the Schiff base bonds between adipic dihydrazide-modified HA and oxidized hydroxymethyl propyl cellulose (as substrates), and incorporated ALG microspheres loaded with the anti-inflammatory drug oridonin and the siRNA-29a gene-loading HA-polyethyleneimine complex. The hydrogel showed suitable mechanical strength and stability, controlled release of the drug and gene complex and in vitro biocompatibility. In vivo, using induced diabetic rats, diabetic wound healing was encouraged by the inhibition of pro-inflammatory factors [89]. Likewise based on the formation of Schiff base bonds, Wei et al. [90] obtained hydrogels by mixing oxidized dextran and antimicrobial peptide-modified and platelet-rich plasma under physiological conditions. In vitro, the hydrogels sustained the proliferation and migration of fibroblasts cells, while in vivo they showed noticeable antibacterial activity, regulated inflammation, inhibited pro-inflammatory factors and accelerated collagen deposition and angiogenesis in a diabetic mouse model [90]. Re-epithelization, a modulated inflammatory response, neovascularization and neoinnervation were promoted in vivo after implantation of stem cell-laden hyaluronic acid-based sponge-like hydrogels into diabetic mice full-thickness wounds [91].

HA was linked with gold nanoparticles in association with photobiomodulation, a useful resource in the treatment of epithelial lesions. Gold nanoparticles alone were efficient in regulating the inflammatory process and modulating the cellular redox state, but used in combination with HA and photobiomodulation, they showed a promising acceleration of tissue repair process by significantly increasing fibroblast activity and collagen production [92].

HA can be successfully used as a cell carrier. For example, Liang et al. [93] explored the potential of an injectable form of HA-tenocytes for the recovery of injured Achilles tendon. Sprague–Dawley male rats suffered single-limb Achilles tendon transection followed by suturing repair. The animals were distributed into three groups and, after surgery, a different solution was injected close to the injured site for each group: HA solution, HA-tenocytes solution and normal saline solution. Then, at certain times, the animals were analysed histologically, mechanically and from the point of view of functionality. The best results were obtained for the HA-tenocytes group, demonstrating an early end of the inflammatory phase and early entry into the proliferation phase.

In another study, HA hydrogel films were studied as in vitro delivery systems for some active compounds, such as peptide fragments. These peptide fragments proved to be promising endogenous inhibitors of elastase. High levels of elastase, along with reactive oxygen species and diminished growth factor activity, were the results of a prolonged inflammatory phase in chronic wounds [94].

### 5.3. Alginate (ALG)

ALG is an anionic polysaccharide extracted from different species of brown algae, a cheap, naturally available and affordable source. In accordance with the source, its structure is based on a varied ratio of guluronate and mannuronate [20], groups with a high Ca content, making it possible for ALG dressings to act as Ca ion donors [95]. ALG is notable for its desirable properties, such as non-toxicity and biocompatibility, but one of the main characteristics that recommend it for wound dressings is its high absorption capacity [20]. As they are widely used in clinical practice, ALG dressings are considered by some authors as a distinct class of modern dressings [96]. Moreover, it is believed that alginate can accelerate the wound healing process by activating macrophages to produce inflammatory cytokines [96]. IN fact, in wound healing, these immune system cells have a key role in the transition from the inflammation to the proliferation phase [97]. These dressings can be used in different phases of wound healing and are adequate for moderate- to heavy-drainage wounds. Due to their bioadhesive properties, they are not suitable for dry wounds and severe wounds, such as third or fourth degree burns. Unfortunately, ALG dressings can dehydrate the tissue and set back the wound healing process, in which case a second dressing is necessary [96].

ALG is used for haemostatic dressings due to its ability to form hydrophilic gels, which absorb wound exudates and interact with cells through bioadhesive bonds. ALG’s crosslinking capacity sustains the exchange of ions with wound fluids, consolidating its haemostatic effect [96,98].

Like other biopolymers, ALG can be processed into a variety of forms. For example, it can be used in injectable hydrogels, which are promising materials for non-compressive wound management treatment [99]. A supramolecular injectable hydrogel with a dense nanofibrillar structure based on a cell adhesive peptide conjugate and ALG, crosslinked with Ca ions and co-assembled, was prepared by Zhai et al. [99]. The hydrogel showed great mechanical properties and increased efficacy in haemostatic control, both in vitro, when spiked with whole blood, and in vivo, in a liver puncture mouse model. Furthermore, the hydrogel encouraged the adhesion and migration of fibroblast cells in vitro, showed demonstrated biocompatibility and promoted the rate of wound healing in vivo in a full-thickness skin defect model in mice [99].

Preman et al. [100] also prepared and characterised an injectable supramolecular hydrogel based on sodium ALG and a synthetic polymer, poly(N-vinyl caprolactam), with haemostatic and antibacterial activity, as confirmed in vitro and in vivo. The hydrogels were made using free radical polymerization and the subsequent chemical and ionic crosslinking. Furthermore, a hydrogel with the same structure, intended for controlled drug release of tannic acid, a natural therapeutic molecule [100], was prepared in parallel. In another study, ALG was mixed with polyvinyl alcohol and Laponite, a smectite clay widely used as a nanomaterial for drug delivery, tissue engineering and regenerative medicine [101]. The authors obtained a nanohybrid interpenetrating network hydrogel with excellent mechanical properties, controlled degradation rate and non-cytotoxic and haemostatic properties, all relevant for wound healing treatments [102].

ALG was successfully mixed with another natural polymer, poly(γ-glutamic acid), to obtain biocompatible porous composite microparticles through an emulsification/internal gelation method. The highly hydrophilic poly(γ-glutamic acid) essentially increases the swelling capacity of composites. In the wound healing field, the composite microparticles have been considered for haemostasis and rapid removal of exudates [103].

ALG has also been processed as sponges, another useful form of wound dressing. A surface-adaptive sodium ALG/gelatine sponge was coated with a polyelectrolyte multilayer film of PHMB and hyaluronic acid, using a spray-assisted layer-by-layer technique. The sponges were biocompatible in vitro and in vivo and showed anti-infection action in vivo when applied in a mouse back infection model and great haemostatic properties, evidenced by a clotting test in vitro and in a liver injury model in vivo [104].

Dowling et al. proved that ALG is also efficient for massive haemorrhages, applying two kinds of ALG-lyophilized sponges, hydrophobically-modified and unmodified, and a commercial gauze dressing (control) to a 6 mm femoral artery puncture in Yorkshire swine that had previously undergone splenectomy. The results suggested that hydrophobic modifications strengthen ALG’s haemostatic ability [60].

Ge et al. published a study in which they prepared and characterized soluble and particulate forms of ALG, and the results indicated that ALG materials have the potential to be applied in inflammation-related diseases [105]. A persistent inflammation can cause infections, poor vascularization and cellular infiltration. For these reasons, dressings for the inflammatory phase should possess antibacterial activity or contain active molecules for controlled delivery [106].

Bioactive nanofibrous dressings based on ALG and lavender essential oil were obtained by electospinning. The dressings had antibacterial effects and stopped the growth of *Staphylococcus aureus* in vitro. The authors also studied the in vitro cytotoxicity of the nanofibres and the anti-inflammatory activity using cytokine expression measurements and qRT-PCR cytokine mRNA quantification. Furthermore, their anti-inflammatory activity was evidenced in vivo in mice with skin burns obtained after UVB irradiation [107].

In another study, ALG was mixed with Aloe Vera gel, another popular natural product with remarkable properties, such as analgesic, antioxidant and anti-inflammatory effects, which are very important for wound healing, as well as the ability to sustain the proliferation of fibroblast and collagen synthesis. ALG and Aloe Vera gel were processed as films with suitable mechanical and physical features for wound-healing applications. A cutaneous wound with a 4 cm^2^ area was made at the dorsum of male Wistar rats, and ALG–aloe films were applied in parallel with ALG films and gauze bandage (as control). ALG–aloe films showed better properties than the ALG films and gauze bandage, modulating the inflammatory phase and reducing the number of macrophages. Another important observation was the enhancement in the evolution of the healing process, suggested by the decrease in type III collagen fibres and the increase in type I collagen fibres [108].

ALG also showed good features for wound healing in combination with synthetic polymers, as in the study published by Salekdeh et al. [109]. Tributylammonium ALG was deposited on polyurethane film, resulting in a biocompatible transparent dressing with antibacterial properties against *Staphylococcus aureus* and *Escherichia coli* and mechanical properties similar to those of human skin. On rats with full-thickness wounds, the dressings reduced the inflammatory phase and improved collagen deposition and the formation of blood vessels [109].

Different polymerization mechanisms were used to manufacture soft ALG hydrogels, which were then ionically and covalently crosslinked, finally resulting in methacrylated ALG hydrogels with tuneable mechanical properties. The hydrogels were sensitive to environment parameters, such as pH, a very important property considering the fact that the pH varies in the wound healing phases (e.g., in the inflammatory phase it is neutral and it changes to slightly basic in the remodelling phase) [110].

Crosslinked superabsorbent ALG hydrogel fibres were used as delivery systems for silver nanoparticles. The complex hydrogel was mechanically robust and biodegradable. Using an in vivo incision wound model produced in hairless SKH-1 mice, the authors demonstrated that ALG fibres loaded with silver nanoparticles are able to reduce the inflammatory phase and enhance epidermal thickness in the repaired wound area [111].

### 5.4. Silk Fibroin (SF)

A brief search in the PubMed.gov platform using the keywords “silk fibroin” and “wound healing” showed 300 research articles and review papers (10%). In contrast to the other biopolymers mentioned above, publications were first recorded in 1998, so its properties are likely not completely acknowledged.

SF has remarkable biocompatibility and a tailorable degradation profile, which make it useful for inclusion in a wide variety of materials for biomedical applications [112,113]. It is a versatile protein that can be processed in different shapes, such as films, electrospun mats, artificial fibres, nanofibrous matrices, hydrogels [114], 3D printed hydrogels [115] and sponges, using various techniques [42,116]. Regarding its applications in skin regeneration, SF showed great properties and influenced the attachment of keratinocytes and fibroblasts, as mentioned by Sun et al. in a recent review regarding the use of SF as a functional biomaterial for tissue engineering [116]. Other important properties of SF that recommend it as an excellent material for wound healing were detailed by Chouhan et al. [117]; namely, it has exudate-absorbing capacities, good mechanical strength (it avoids wound bed disruption), good elasticity (it can conform to the wound size and shape), good wound healing action (it helps in neovascularization, enhanced re-epithelialization and tissue ingrowth), it can be easy functionalized and it is cheap (due to the affordable natural source).

For use in anti-adhesion barriers and antithrombotic materials for wound healing, SF is frequently combined with synthetic materials, such as N, N′ methylene bisacrylamide [118], polyurethane [119] and poly(vinyl alcohol) [120].

Poly(ethylene glycol) (PEG), another synthetic polymer, is often mixed with SF, resulting in a gel composed mainly of a β-sheet structure [113]. PEGylated SF films were proved to be useful anti-adhesion and antithrombotic materials thanks to their ability to modify cell interactions related to adhesion and proliferation [112].

Wei et al. [113], in a research article published in 2020, observed that SF plays an interesting role in the blood coagulation process. The authors combined SF with PEG and obtained a sponge that promoted platelet adhesion and aggregation, as well as platelet-fibrinogen interaction, in vitro, features that recommend it as a haemostatic material.

Histological examination and molecular assays have shown that a bilayer skin substitute based on human amniotic membrane and electrospun silk fibroin nanofibres supported epidermal and dermal regeneration in a murine full-thickness wound model [121]. The same composition was used to prepare 3D protein-based artificial skin implanted on full-thickness burn wounds in mice. The application showed promising results for the targeted application: stimulating wound-healing process and guiding the healing of severe burns without scars [122].

### 5.5. Collagen (Col)

Col, an extracellular matrix component, is the most abundant protein in the human body, and it has crucial roles in sustaining and maintaining the tensile strength of tissues. Often used as a biomaterial in fields such as tissue engineering and regenerative medicine, including wound healing, this biopolymer has incredible physical and biological properties, such as biocompatibility, biodegradability, enhancement of new collagen deposition, haemostatic abilities and control of proteolytic activity in chronic wounds. These properties are influenced by the main sources, which may be human tissue or that of animals, such as cattle, pigs, sheep and even fishes, and also by the tissue from which it is isolated. Like other biopolymers, collagen can be easily manufactured in different forms: fibres, scaffolds and hydrogels [123].

In addition to its well-known properties, Col has an important role in blood clotting. After the platelets come into contact with collagen, a reaction cascade starts. First, the aggregation of platelets occurs, which is followed by the formation of a clot, resulting in reduction of bleeding [123].

Kumar et al. reported use of a nanofibre-forming collagen mimetic peptide as a synthetic biomimetic matrix, with applications in thrombosis treatment. Platelet adhesion, morphology and activation, plasma and whole-blood clotting kinetics and haemolytic and pro-inflammatory potential were studied in vitro, and the results demonstrated the potential of collagen material as a haemostat [124].

Using a specific gradient-removal solvent approach, a Col aggregate/chitin-based biomaterial with a biomimetic 3D microstructure, “cotton-like” appearance and superior thermo-stability, biodegradability and biocompatibility (proved in vitro in L929 fibroblast cells and in vivo in Sprague–Dawley rats) was obtained. Haemostatic performance was first evaluated in vitro, by measuring the blood clotting index and total coagulation time, and then in vivo in three animal models: a rat muscle traumatic haemostasis model, a rat liver wound haemostasis model (Sprague–Dawley rats) and an adult, healthy, Japanese white rabbit central ear artery traumatic haemostasis model. The results of the studies indicated the potential use of the biomaterial for functional haemostatic materials [125].

Col and another hydrophilic biopolymer, γ-polyglutamic acid, were crosslinked with water-soluble carbodiimide, resulting in a gel with advanced viscosity and excellent mechanical strength. The gel was implanted in a dorsal bilateral skin incision in Wistar rats and showed gradual degradation, promotion of cell adhesion and facilitation of the migration of fibroblasts in its structure and maintenance of the reconstituted collagen fibrils and haemostasis, with all these indicating it as a promising scaffold for tissue repair [126].

### 5.6. Combination of Biopolymers

The treatment of diabetic wounds is a real challenge, mainly due to the fact that the prolonged phase of inflammation obstructs the further phases of healing. Chitosan/collagen scaffolds have been used by hundreds of researchers for 25 years, mainly for wound healing but also for spinal cord injury, osteochondral tissue repair, bone and periodontal tissue engineering and so on. Composite scaffolds based on the two polymers and loaded with a pioglitazone-nanostructured lipid carrier were prepared and studied for their potential healing ability in diabetic wounds by Natarajan et al. [127]. After several characterizations that proved that the scaffolds possess adequate porosity and controlled degradation and drug release, the in vitro assay verified its biocompatibility and capacity to enhanced cell growth. In vivo, in a diabetic wound model with male Wistar rats, an enzyme-linked immunosorbent assay indicated a significant decrease in matrix metalloproteinases-9 levels [127].

A three-layer nanofibre biopolymeric wound dressing, loaded to the shell with doxycycline, was evaluated for its antibacterial and anti-inflammatory properties from histological, biochemical and immunohistochemical points of view using a full-thickness wound model in normoglycemic rats (acute model) and diabetic rats (chronic model) in comparison with a commercial product. The core layer of the dressing contained collagen, while the other layers included chitosan and alginate. Immunohistochemical assays revealed decreased levels of matrix metalloproteinase enzyme and increased levels of tissue inhibitor of metalloproteinase, which were attributed to the use of the antibiotic and also the fact that the dressing improved angiogenesis and shortened the inflammatory phase. The biochemical assays proved the dressing’s effectiveness in the inflammation and proliferation phases, while the immunohistochemical and histological assays highlighted the dressing’s strength and safety when using it for both acute and chronic wounds, demonstrating similar effects as the commercial dressing [128].

Cs–ALG membranes have been studied as wound dressings by different researchers [129,130]. The mixture of the two polysacharrides resulted in a crosslinked polyelectrolyte complex with superior features, such as structural strength, mechanical stability and swelling capacity [130]. Studied in a cutaneous wound model in Wistar rats, the membranes showed inflammatory cell recruitment even from the second day after implantation. After 21 days, the authors concluded the study with encouraging results: the membranes modulated the inflammatory phase, encouraged fibroplasia and collagen genesis and, in the earlier phases, intensified cutaneous wound healing. Moreover, it was observed that the membranes enhanced the quality of the scar tissue and were non-adherent [130]. In mice with diabetes mellitus induced by streptozotocin, the inflammatory phase of the cutaneous wound was modulated using membranes based on the same biopolymers mentioned in the previous study. Ten days after injury, complete re-epithelialization with appropriate organization of collagen fibres and a decrease in inflammatory infiltrate cells was observed [129].

## 6. Emerging Therapies Using Biopolymers

**Figure 2:**
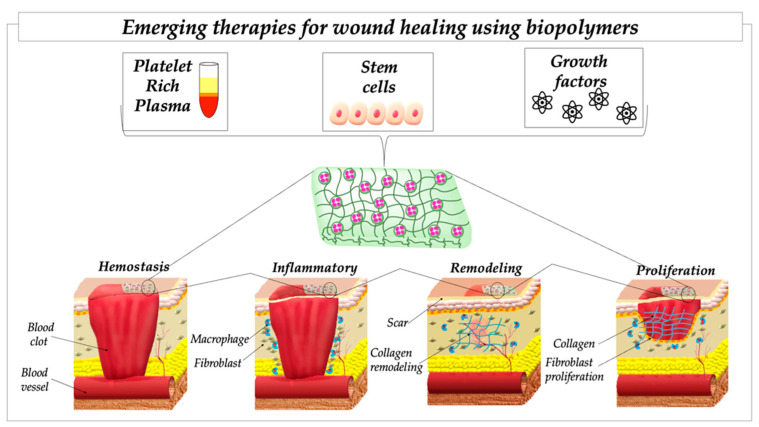
Emerging therapies for wound healing using biopolymers.

Although the existing research has confirmed that biopolymers play positive roles in different shapes and structures in all phases of the promotion of wound healing, the emergence of various biological agents, such as stem cells, PRP and monoclonal antibodies, either alone or combined with polymer carriers, has led to the gradual replacement of traditional dressings. In this section, we provide an overview of the possible ways that nature could provide inspiration for more exciting progress in the future of healing (Figure 2).

### 6.1. Blood Products: Platelet-Rich Plasma

PRP, a prominent blood-derived product, has nurtured a significant level of appreciation since its first occurrence around the 1970s among medical experts, including oral and maxillofacial surgeons, dermatologists, ophthalmologists, gynaecologists, urologists, cardiologists, paediatric and plastic surgeons and veterinarians. The term “platelet-rich plasma” describes the plasma isolated after a two-step centrifugation process with a platelet count about three to five times the concentration of platelets found in whole blood. According to Alves and Grimalt’s [131] review from 2018, the supraphysiological platelet concentration found in PRP is higher than 150,000–400,000 per cubic μL. PRP is rich in various proteins and growth factors, including platelet-derived growth factor (PDGF a-b), transforming growth factor (TGF-β1), epidermal growth factor (EGF), insulin-like growth factor (IGF-I, IGF-II), fibroblast growth factors (FGFs) and vascular endothelial growth factors (VEGFs), as well as cytokines, serotonin, adenosine, dopamine, histamine and calcium. More details about PRP-based growth factors and platelet cytokines, along with their cell sources and their specific functions, can be found in Everts et al.’s [132] review. There are numerous review papers dealing with PRP’s preparation, performance and applications, starting from the first publication in 2006 [132] focused on the developments regarding PRP preparation and composition, mechanisms related to inflammation and angiogenesis in tissue repair and regenerative processes and, lastly, the effect of certain drugs on PRP activity and the combination of PRP and rehabilitation protocols.

In 2013, Andia and Abate [133] defined the basic science of healing with PRPs, along with the link between the biological mechanisms influenced by PRP and the clinical correlates. Their review emphasizes the fundamental idea behind PRP therapies, which have so far been applied to thousands of patients but which still have limited response rates for precise indications, insufficient evidence of significant clinical improvements and problems with health insurance reimbursement. The authors propose in the section on future perspectives that a step forward in the success of PRP therapies would be the discovery of predictive biomarkers to identify PRP responders.

The book chapter by A. Lehn [134] from 2020 introduces the commitments of PRP in tissue engineering and regenerative medicine. After the introductory section related to PRP composition and its mechanism of action, the different protocols used to obtain PRP products from whole-blood samples are presented in detail. As expected, the author highlights the variety of protocols available to obtain PRP products and the fact that only a few allow for quantitative composition; therefore, intensive research is essential for the development of standardized protocols. The author also dedicates an entire section to the application of PRP-based biomaterials in tissue engineering and regenerative medicine, describing several experiment-related articles and highlighting the necessity for clinical trials in order to establish the conditions under which the PRP should be properly and precisely employed.

As already discussed in numerous papers, PRP can be obtained by using various available protocols, either via commercial kits or manual techniques, the latter demanding two centrifugations with specific centrifugal force (g) parameters and a predefined time. Typically, the erythrocytes are separated from the plasma and the buffy coat (the delicate layer of leukocytes on top of the erythrocytes) after the first centrifugation step. Then, the plasma, with or without the buffy coat, is centrifuged to concentrate the platelet, resulting in platelet-poor plasma and PRP at the bottom layer of the suspension [135]. However, although there are many different protocols available, there is no ideal method for the PRP preparation, which creates variance in the final concentration of the components, the constituents’ nature, the number of active platelets, the efficiency rate, the ideal administration volume, the frequency of application, the exact site of administration and so on.

In the future, the objective of researchers should be to ensure that all these issues are resolved and that PRP technology lives up to its promise based on the available data. Therefore, in 2020, Andia et al. [136] proposed freeze-drying as a consistent method for PRP product standardization and fabrication with improved stability, making these products ready for future uses. The systematic search revealed that, among the 46 included articles, skin research and wound management occupied the first place with 32 papers, followed by musculoskeletal conditions (10 studies) and dentistry (7 studies). Different PRP formulations have so far been lyophilized, either from anticoagulated peripheral blood or from peripheral blood and with single or double spinning, resulting in products that preserve platelet integrity (PRP, platelet concentrate, platelet-rich fibrin), as well as lysates and releasates, mostly composed of platelet secretome (from either PRP or platelet concentrates).

Furthermore, although the literature on PRPs has developed intensively, there are still numerous contradictions in the classification terminology. The most complete classification was proposed in 2016 by Magalon et al. [137], based on dose, efficiency, purity and activation parameters. The so-called DEPA classification focuses on the platelet concentration obtained with PRP kits, the purity of the PRP obtained, the efficiency of the production and platelet activation prior to injection. However, as expected, this is not a standardized classification or characterization method for PRP, since it does not consider whether or not cells are applied live when the PRP is not activated [131], a necessary element for clinicians when selecting a system to meet their specific needs for a given indication. In 2018, the International Society on Thrombosis and Haemostasis (ISTH) proposed for the first time, at the Scientific Standardization Committee (SSC) meeting, recommendations for standardizing the use of platelets in regenerative medicine [138]. The ISTH comprehensive classification takes into consideration sample collection conditions (coagulated or not), the preparation method, WBC and RBC content, the number of platelets, platelet enrichment and activation and whether the PRP product has been frozen/thawed prior to use.

Acebes-Huerta et al. [139] proposed a matrix for a new nomenclature classification of PRP products based on the information available in 2020. This tentative, new nomenclature system defines the subscript, superscript and prefix parameters that refer to the extraction method, processing procedures prior to application, characteristics and quality of the product. More details can be found in their review. Furthermore, the authors presented an ideal shape for PRPs, recommended as a “Trojan Horse” for loading drugs or biological therapies, and, considering all the above-mentioned features, they stated that the WBC volume should be lower than 10^−5^–10^−6^/unit, with no RBC contamination.

After successful implementations in dental and maxillofacial surgery and in the musculoskeletal field for sports injuries, the interest in the application of PRPs for tissue regeneration, wound healing, scar revision and skin rejuvenation has been boosted in the last decade [140] due to their favourable wound-healing, haemostatic and angiogenic features.

A recent review by dos Santos et al. [141] exploits the role of autologous PRP-derived components in the application of regenerative medicine, stating that, although many investigations have demonstrated its supportive therapeutic effect in tissue healing, further studies are still necessary. After a brief introduction to PRP evolution and the current protocols, the authors discuss the implications for inflammation, cytokine profiles and anabolic and fibrinolytic cascades, shedding light on the known PRP mechanisms. The application of PRP derivatives degranulates and releases the relevant growth factors and proteins for an extended amount of time, simulates and supports the physiologic wound-healing microenvironment, modifies cellular recruitment of inflammatory cells and MSCs, regulates proteolytic activity and recruits more cell types, inducing the acceleration of the healing cascade. Therefore, even if PRPs can be considered an ideal biological tool due to their low cost and minimal risk, this tissue-healing agent remains complex, mainly since it delivers a broad range of bioactive factors with interactive mechanisms that have not been completely elucidated.

The recent review by Everts et al. [142] also outlines the feasibility of generic PRPs in mimicking the initiation of the healing cascade, defining them as living biomaterials with clinical outcomes dependent on the patient’s blood complex constituents and the interaction with the local microenvironment.

In Table 3 and the text below are highlighted some representative studies concerning different products based on PRP and biopolymers.

Rossi et al. [145] developed a novel dressing for chronic skin ulcers by combining delivery of platelet lysate (PL), rich in growth factors capable of promoting tissue regeneration, and vancomycin hydrochloride (VCM), an anti-infective model drug. The circular dressings were fabricated using a simple method by dropping a mixture of 6% hyaluronic acid (HA) aqueous solution containing 1 or 2% CaCl_2_ and PDGF a-b into 1% sodium alginate (ALG) aqueous solution containing VCM (3 mg/mL), which was stirred and subsequently freeze-dried. The dressings displayed suitable mechanical, hydration and rheological properties. High loading capacity and release were observed for VCM independently of CaCl_2_ concentration, since VCM was loaded in the alginate matrix and not in the HA particles. The loading capacity for the PDGF a-b was influenced by the CaCl_2_ concentration; the highest amount was found in the 2% formulation due to the higher degree of crosslinking. As expected, the release profile showed that the highest concentration of CaCl_2_ resulted in a lower amount of PDGF a-b released, an amount that increased in time. The cell proliferation assays indicated the formulation’s ability to enhance fibroblast proliferation due to the release of PDGF a-b in comparison to the unloaded formulation, results sustained by the assessment of DNA synthesis by BrdU incorporation. An important contribution of the authors was the ex vivo tests in a human skin biopsy, undertaken to analyse the effects of dressings on wound healing. The microphotographs of haematoxylin and eosin-stained sections of human skin biopsies after 72 h treatment with the loaded dressing showed an active regeneration process, with collagen bundles oriented according to evident structural planes.

PRP was also loaded by Wang et al. [151] in 2019 onto a composite scaffold based on chitosan fibroin emulsion with added silver nanoparticles (AgNPs), using freeze-drying technology to prepare the tissue engineering dressings. The results showed the asymmetric wettability of the composite dressing, along with relatively high porosity, favourable moisture retention, appropriate tensile strength and satisfactory antibacterial properties. The good biocompatibility and lower sensitization resulted in increased healing efficiency for the infected wounds examined in mice. The satisfactory wound tissue repair and regeneration profile were sustained by bacterial cultures of the wound exudate, whole blood cell analysis and histological examination.

Samadian et al. [143], in 2020, developed an artificial scaffold based on a polycaprolactone/gelatine nanofibrous nerve guide conduit (NGC) filled with citicoline-bearing PRP gel as an effective tissue engineering treatment for peripheral nerve injury. The fabricated nanofibres showed favourable structural features; namely, suitable mechanical strength from PCL, numerous cell attachment sites from gelatine, anatomical regeneration and functional recovery of the damaged nerve capability due to citicoline and beneficial biological properties from PRP. The in vitro release profile of the NGC exhibited a sustained release of citicoline of about 50% within 14 days. The in vitro blood compatibility and cell proliferation tests revealed that the nanofibres were haemo- and biocompatible. After the implantation of the citicoline–PRP-loaded NGC for sciatic nerve injury in adult male Wistar rats, the various studies conducted, such as walking-footprint analysis, a hot plate latency test, the gastrocnemius muscle wet weight recovery ratio and a histopathological examination, indicated enhanced nerve cell growth, regenerated nerves and acceptable functional recovery.

In the same year, Nardini et al. [148] developed an advanced wound dressing based on the combination of alginate (ALG) and sericin (SS) with platelet lysate (PL) in the form of a freeze-dried sponge, capable of tuning cell behaviour and promoting growth tissue in the early stages of wound healing. The high release level for platelet growth factors (at 48 h), minimum cytotoxicity, the capacity to protect cells against oxidative stress and cell proliferation induction revealed the potential of this dressing for testing in a mouse skin lesion model. The applied biomembranes led to faster regeneration of the skin wound compared to the control due to an accelerated burst of granulation tissue, early inflammation, collagen deposition, fibroblast maturation, re-epithelialization and neovascularization. It is important to mention that this was the first study on the regenerative effect of an SS/PL-based dressing on wound healing in vivo.

In the study by Qian et al. [154], poly(ethylene glycol) (PEG) functionalized with 4-carboxybenzaldehyde was used as a crosslinker for glycol chitosan hydrogel formation. Silk fibroin and PRP were then loaded into chitosan-based hydrogels with a mixing/freeze-drying method, resulting in the formation of an injectable, physically stable and self-healing hydrogel. The performed characterization and the in vitro and in vivo tests demonstrated the product’s resistance to enzymatic hydrolysis and biosafety, as well as its capacity for sustainable release of PRP and repair in treating a diabetic full-thickness skin injury in a type 2 diabetes rat model through stimulation of neurogenesis and vasculogenesis.

A new composite wound dressing comprised of rhizochitosan and platelet concentrates was developed by Chen et al. [144]. First, rhizochitosan composed of 13.33% Cs was obtained by deacetylation of rhizochitin through an efficient and economical means of fabrication; namely, depigmenting a sporangium-free mycelial mattress from *Rhizopus stolonifer* F6 in potato dextrose broth, without requiring bleaching. The porous fibrous network incorporated different concentrations of the commercial product Regenplex™, which consists of PRP powder, and the product was then freeze-dried in moulds of various forms to obtain appropriately sized wound dressings. The in vitro release profile was evaluated after suspending the platelet-fused network in saline solutions and the results indicated that TGF-β was released mainly during the first week and PDGF between days 8 and 16, and the amounts decreased gradually for up to 32 days, as determined by immunoassay kits. Then, the sponge mattresses were tested as wound dressings in a full-thickness wound model in male Sprague–Dawley rats. The photos of the wound closure and the results showed that the composite dressing significantly reduced the wound area, more notably than the individual components alone. The same promotion of wound healing effects was observed at the histological evaluation with H&E staining at 14 and 21 days. The authors also evaluated gelatine zymography during the wound-healing process, using MMP-9 and MMP-2 as standards. The tests revealed that MMP-9 expression completely disappeared by days 14 and 21, and MMP-2 expression showed the opposite trend, being barely observable on day 1 and significantly increased in days 7 to 21.

The addition of PRP and gentamycin sulphate into the biocompatible wound dressings based on carboxymethyl chitosan and gelatine microspheres, as prepared by Shi et al. [155], enhanced the granulation stage and reduced the inflammatory period of *S. aureus*- and *E. coli*-infected full-thickness skin wounds. As well as the usual physiochemical features of the proposed dressing, such as increased water uptake ability, suitable porosity, good mechanical properties and sustained release of PRP and antibiotics, the in vivo studies on Sprague–Dawley rats revealed accelerated re-epithelialization, collagen deposition and angiogenesis of the wounds.

The list of biopolymers and PRP could continue since there have been remarkable developments in the use of platelet-based products at the preclinical stage, but clinicians are still sceptical regarding their use. As mentioned above, the variability in the products and the methods for obtaining them, the different nomenclatures and the safety data are among the issues most reported by clinicians, as well as patients. However, the bright side of this matter is that, so far, there are few reported adverse effects from the use of PRP in therapy; therefore, we strongly encourage clinicians to perform studies using PRP-based products in order to fully describe the types and the relevant methods required to obtain proper characterization and expand the application field in regenerative medicine.

The clinical benefits of PRP in wound healing are being investigated in several registered clinical trials (available at www.clinicaltrials.gov, accessed on 30 September 2021), as briefly described below.

In 2009, the Centre Hospitalier Universitaire Vaudois, Switzerland, submitted an interventional randomized trial with parallel assignment, identified as NCT00856934 [157], that evaluated, from 2005 up to 2006, a new model for autologous keratinocytes suspended in platelet concentrates, aiming to enhance healing on skin graft donor site wounds. The study enrolled 45 participants divided in three arms: patients with control wounds covered with standard dressings (experimental group 1), patients with PRP sprayed onto the wound bed with calcium chloride (experimental group 2) and patients with keratinocytes suspended in PRP sprayed onto the wound bed with calcium chloride, with both the latter groups’ wounds being covered with standard dressings. The results for the main outcomes showed that, for the control group, the time required for complete epithelialization was 13.9 days, for it was PRP 7.2 days and for keratinocytes and PRP it was 5.7 days. Furthermore, the same trend was observed for the second outcome results; namely, the pain experienced during the dressing replacement as evaluated on a visual analogue scale (VAS) from 0 (no pain) to 10 (extreme pain). The control group obtained a score of 7.2, while the PRP 1.5 and keratinocytes and PRP groups obtained scores of 0.4. Overall, the study demonstrated that the addition of PRP into the treatment sped up and improved wound healing.

The role of autologous PRP in total knee arthroplasty was evaluated in an interventional randomized clinical trial (NCT01563380) [158], which started in 2010 and was completed in 2012. The main hypothesis of the investigators was based on the potential of PRP application for reduction of blood loss and postoperative pain and acceleration of wound healing following total knee arthroplasty. The enrolled patients (40) were divided into a control arm and PRP arm, and the PRP was applied over the wound, including the capsule and medial and lateral recesses. The results have not been published yet, but the main investigator published an article in 2014 in which the positive effects of PRP were presented [159]. The authors described a significant reduction in blood loss and pain and the need for narcotic use after application of the PRP but an insignificant difference in the wound-healing rate between the groups.

Another trial that begun in 2018 and was completed in 2021, identified as NCT04697082 [160], evaluated the impact of autologous PRP on the wound-healing rate, on pain reduction and on quality of life before the surgery and three weeks after compared to minimally invasive techniques. The 63 enrolled patients were randomly divided into three groups as follows: group A—control (open healing and application of saline solution), group B (open healing and application of PRP) and group C (application of PRP after curettage of the sinus cavity). The results of the study have not been published but, according to a recent related article by the investigators [160], only 49 patients with pilonidal sinus disease remained in the study and they were evaluated according to the trial design. Several aspects of the study were notable: the cavity volume and wound-healing time did not differ between groups A and B, but the recovery time per unit of cavity volume was significantly faster in group B than in group A, and better than in group C. There was no significant difference for the VAS scores on day 1, but the scores started to decrease from day 2 to 5, and there was a reduced need for painkillers. The scores for the quality of life of the tested groups were insignificant in the preoperative/postoperative period but improved in group B when the pain and general health perception parameters were considered in isolation. Overall, despite the occurrence of abscesses in four patients from group C, after the fifth PRP dose was applied, postoperative recovery was improved, pain was reduced and quality of life increased for patients with pilonidal sinus disease.

The clinical trial identified as NCT03085550 [161], completed in 2020, aimed to investigate the feasibility of conducting a randomised controlled trial with fat grafting and fat/PRP co-grafting as interventions for diabetic ulcers. The investigators evaluated the rate of wound healing in diabetic ulcers when treated with conventional dressings, fat grafting and combined fat and PRP. This trial, estimated to include approximately 30 patients (three arms) and conducted at the Royal Free Hospital and UCL Division of Surgery and Interventional Science, followed and assessed for 12 weeks the rate and degree of wound healing and evaluated the mechanism of healing through histological analyses at day 0, week 1 and week 4. The use of the fat and PRP mixture as a treatment, which demonstrated improved healing, shorter follow-up and a reduced cost burden, was presented in two recent publications by the investigators. In the first, Smith et al. [162] identified that the proposed randomised controlled trial was feasible and safe. The second paper evaluated the clinical outcomes (wound size and healing, adverse events, cost implications, etc.) and health-related quality of life (questionnaire). The article also highlighted the key barriers for recruitment, study design and protocol execution, outcomes and data collection for a larger multicentre trial, but it could not assess the effectiveness of the fat and PRP treatments, probably due to the small number of included patients.

The second publication aimed to correlate the histological changes from the fat grafts and PRP treatment for diabetic foot ulcers [163]. In order to establish this, the investigators analysed 18 diabetic foot ulcer patients divided into three arms: fat grafting, fat grafting with PRP and a control group. At weeks 0, 1 and 4, punch biopsies were obtained from the centre and edge of the patient wounds after the initial intervention and stained with haematoxylin and eosin for microarchitecture analysis, CD31 for microvessel density analysis and Ki67 for cell proliferation analysis. Based on the obtained results from the combined fat and PRP arm—namely, large volumes of organized mature adipocytes, enhanced fat graft survival and increased microvessel density at 1 week post-treatment—the study demonstrated that PRP with fat grafts positively affected the histology of diabetic ulcers. However, the visual approach used to measure fat graft survival had some limitations; therefore, future studies using apoptosis markers or florescent labelling are necessary to ascertain the occurred mechanism. The authors located a similar limitation for the increased microvessel density, since it was not clear which cell type was responsible for this. The proposed solution was that VEGF staining might explain the unanswered question. The article concluded that a future trial should enrol a suitably powered study population.

When the PRP and wound healing keywords were used in the search engine of ClinicalTrials.gov, the platform listed 37 registered trials, among which several have been described above; but, when we added biopolymers, no studies were found. From the perspective of a polymer specialist, this could also bring additional advantages in wound-healing management. A noteworthy clinical study that could sustain this hypothesis was published by Goyal et al. [153], who selected 30 patients with periodontitis and apicomarginal defects and evaluated a commercial collagen sponge with PRP for their tissue regeneration. The obtained results displayed high healing rates of 83.33% and 88.89% for PRP and PRP combined with the collagen sponge, respectively, compared to 80% in the collagen membrane arm according to the clinical and radiographic controls, performed every 3 months over a period of 12 months. Although the authors stated that PRP may be used as an alternative treatment for apicomarginal defects, there were still limitations: a histological assessment was used to confirm the efficacy of the periodontal grafts since clinical and radiographic analyses do not indicate regeneration, the study had low statistical power and regeneration difference due to the variability in defects and the fact that long-term studies would be required to generalize the outcomes.

It is important to mention that platelet therapy represents a different perspective from growth factor therapy since platelets contain the necessary growth factors that regulate haemostasis, fibrin clot formation and tissue repair. By association, its use has low risk, as an autologous biological product derived from patient blood represents a cost-effective alternative for different types of wound healing (see Table 1 for more features).

### 6.2. Therapies Based on Stem Cells

Therapies based on different types of cells have been proved to be able to accelerate wound healing and even to regenerate affected tissues, preventing wound contracture or scar formation [164,165]. Stem cells, characterized by the capacity for self-renewal, asymmetric replication, differentiation into other types of cells, unlimited replication and the capacity to secrete pro-generative cytokines, represent a very promising strategy for use on a large scale for wound healing [166,167]. Among the main sources of cells that might be used for wound healing and regeneration of injured skin are embryonic stem cells (ESCs), induced pluripotent stem cells (iPS) and adult stem cells, mesenchymal stem cells, adipose-derived stem cells and hematopoietic stem cells [168].

Modern reconstructive medicine and tissue engineering are based on the extraction and separation of cells from living tissue, in vitro cultivation and expansion and their encapsulation in a 3D scaffold in order to avoid the intervention of the immune system and, finally, to repair damaged tissue [169].

Cell-based approaches to wound healing can use cells embedded into various materials or deposited on different scaffolds, with the aim of assuring physical contact with the wound area, followed by the proliferation, differentiation and/or migration of the cells into the injured area and, finally, regeneration; alternatively, they could be used as a supply pool for different growth factors that can accelerate the healing.

In order to prevent the immune system attacking transplanted cells, cell encapsulation is a very versatile and useful technique that allows the bidirectional exchange of oxygen, nutrients, waste products and therapeutic proteins between host and encapsulated cells [170], a technique that involves the localization of intact cells to specific regions in a device or material without the loss of the requisite biological function. Immobilization of cells can generally be performed through physical adsorption, encapsulation, entrapment and self-aggregation [171].

A faster skin wound-healing rate with greater neovascularization was obtained using human umbilical cord mesenchymal stem cells (hUCMSCs) cultured with alginate gel scaffolds in an experimental model. The hUCMSC–alginate gel mix was transplanted into Balb/c mice skin defects to investigate the healing rate and immunohistochemistry markers. The wound healing was faster in the hUCMSC–alginate gel-treated group compared to the control group, the improvement in the healing rate being achieved through paracrine signalling [172].

To improve the success of healing, not only 2D cell cultures but also 3D cells were embedded in different scaffolds, these latter being more physiologically similar to normal conditions in different tissues. Thus, the hydrogel–3D mesenchimal stem cell spheroid (3D MSC) combination therapy accelerated wound closure in diabetic mice. In this case, 3D mesenchymal stem cell spheroids, which were mixed with thermosensitive chitosan/collagen/β-glycerophosphate (β-GP) hydrogels and rapidly transformed into a gel at body temperature, fitted any wound shape. The hydrogel assured an adequate environment for the attachment and proliferation of encapsulated mesenchimal stem cells, accelerating the proliferation and paracrine factor secretion of 3D MSC spheroids, finally assuring tissue remodelling via physical or chemical factors [173].

Furthermore, good results were obtained by using an adipose tissue-derived injectable extracellular matrix/stromal vascular fraction gel (ECM/SVF-gel), which was applied in a nude mouse excisional wound-healing model. The effect of this combination was in vitro promotion of the angiogenesis (tube formation) and increased expression of the angiogenic factors vascular endothelial growth factor (VEGF) and basic fibroblast growth factor (bFGF) compared to those in the control. However, the expression of the inflammatory chemoattractant monocyte chemotactic protein-1 (MCP-1) was high in the ECM/SVF-gel at the early stage, but its expression decreased during the late stage of wound healing. Mesenchymal stem cells are an attractive cell type for cytotherapy in wound healing [174].

Nilforoushzadeh et al. [175] reported the use, in a clinical feasibility study, of autologous dermal-epidermal skin grafts with intrinsic vascular plexus transplanted into human subjects (five patients) with diabetic wounds and compared with nonvascularised skin grafts as the control (five patients). Adipose-derived stromal vascular fraction (SVF) cells were mixed with a hydrogel fabricated from fibrinogen and collagen and transplanted into patients’ wounds after checking the moisture balance, inflammation and infection control. The obtained data indicated that autologous SVF could be used to construct epidermal-dermal skin-like compartments and create a prevascular network in the dermal layer based on hydrogel grafts, and they were safe and able to accelerate the wound-healing process.

Another approach for the treatment of difficult wounds could be based on the use of exosomes derived from stem cells embedded in hydrogels. A constructed combination of human umbilical cord MSC (hUCMSC)-derived exosomes (hUCMSC-exos) and Pluronic F-127 (PF-127) hydrogel was topically applied to a full-thickness cutaneous wound in a diabetic rat model. This experimental biomaterial-based exosome therapy showed a significantly accelerated wound-closure rate, increased expression of CD31 and Ki67, enhanced regeneration of granulation tissue and upregulated expression of vascular endothelial growth factor (VEGF) and factor transforming growth factor beta-1 (TGFβ-1). The PF-127 hydrogel was fully compatible and degraded rapidly in in vivo conditions, whereas exosomes promoted the vascularization of wound granulation tissue, shortening the diabetic wound-healing time [176].

Palakkara et al. [177] evaluated the healing potential of a porcine decellularized small intestinal submucosa (SIS) matrix and chitosan with mesenchymal stem cells (rMSC) and epidermal growth factor (mEGF) in a full-thickness burn wound model in rats. The authors created one 2 × 2 cm^2^ sized full-thickness burn wound on the dorsum of each animal and treated them with silver sulfadiazine as the control group, medical-grade chitosan powder, chitosan powder with rMSC, chitosan powder with rMSC and mEGF and decellularized porcine SIS matrix, as well as rMSC seeded with SIS and rMSC seeded with SIS and mEGF. Based on the percentages of wound contraction, colour digital imaging and immunological, histopathological, immunohistochemistry and gelatine zymography observations, the decellularized SIS matrix combined with the r-MSC and m-EGF accelerated angiogenesis and epithelialisation. Furthermore, the combination of rMSC and mEGF with chitosan powder was found to have good healing potential but was not as effective as the SIS seeded matrix, this latter being considered the best treatment for repair of full-thickness burn wounds in a rat model.

### 6.3. Growth Factors

So-called “bioactive dressings”, based on growth factors integrated into different biomaterials, have been widely applied for tissue regeneration. As explained in-depth by many review papers, the wound-healing process involves a complex cascade of cellular and biochemical events. Depending on the timeline of the healing phase, different platelets, neutrophils, monocytes/macrophages, fibroblasts, lymphocytes, granulation tissue cells and epidermal cells appear at the “crime scene”. These cells release a series of biological molecules, among which are growth factors, which have an important role in wound healing.

Topical products containing growth factors, such as platelet-derived growth factor (PDGF), basic fibroblast growth factor (bFGF) and epidermal growth factor (EGF), are already approved and available on the market [178]. Their use in the clinic confirms the importance of these molecules for wounds. However, the delivery of growth factors from creams, gels or ointments is not effective enough for chronic wounds; therefore, they are combined with secondary dressings, increasing the cost of therapy.

The use of naturally derived polymer-based supports (micro/nano-sized) for the loading of single or multiple growth factors exhibits several advantages: high loading capacity, simultaneous loading/delivery of multiple growth factors, controlled release, biocompatibility and low side effects. However, while all these benefits are well-established at the laboratory scale, there is limited research on their use in clinics, mainly due to the high costs and lack of quality control.

The review by Catanzano et al. [179] discusses, in detail, the design, characterization and evaluation of wound dressings loaded with growth factors. As expected, many papers have been published, as described in the above-mentioned review, that highlight the potential of integrating growth factors into biopolymer constructs for wound dressings. The authors present specific examples of such platforms based on natural polymers, such as gelatine, alginate, collagen, chitosan, cellulose, fibrin, dextran, hyaluronic acid and their derivatives and combinations, that have been investigated up to 2020. For the next year (2021), a brief search on the PubMed platform using the terms “growth factors”, “biopolymers”, “wound” and “healing” returned nearly 127 results, but only a few were considered relevant for this review paper, as briefly presented in Table 4.

As observed, the biopolymers used to incorporate growth factors were processed in the form of hydrogels, scaffolds, nano/microspheres and nanoparticles, films, etc.; substrates that, besides their obvious release capability, had synergistic effects on wound healing by promoting cell adhesion and proliferation. The growth factors were loaded into the biopolymer carrier systems based on their affinity, interactions, encapsulation efficiency, stability and controlled release into the wound setting. These parameters ensured the increased bioactivity of the growth factors, making them suitable for wound-healing functions.

Considering the amount of research in the area of wound healing dressings based on growth factor enrichment, it is obvious that these “bioactive dressings” will be utilized in routine wound care management. Since, for the moment, the major drawback is the high cost, it is expected that, in the next decade, a well-defined mixture of growth factors and biopolymers will become cheaper and safer substitute for skin grafts. Moreover, considering the current trend for personalized clinical care, genetic variability, wound types and the patient’s clinical and metabolic features will be considered in order to offer specific and more effective therapies.

## 7. Conclusions and Future Outlook

The progress of intelligent regenerative approaches towards wound healing will continue to grow in coming years, with particular attention being paid to bioactive potentialities and sustainability. Biopolymers, credited as being highly biocompatible, biodegradable and with anti-inflammatory, antimicrobial, haemostatic, cell proliferative and angiogenic activities, have been explored for centuries in the fabrication of wound dressings. Currently, numerous studies report that different derivative of biopolymers—namely, ALG, cellulose, Cs, Col, HA and SF—have already been applied as wound dressing vehicles in clinical practice. The favourable results achieved can also pave the way towards wound healing based on interdisciplinary approaches without any undesired outcomes. A promising strategy in the treatment of wounds is the use of biopolymers coupled with blood products, growth factors or cells that mimic synergistic healing events and activate essential regenerative pathways beneficial to wound healing. Thus, these sophisticated platforms inspired by nature will improve and accelerate the wound-healing process and provide great benefits for patients in the near future.

However, the question is how future generations of wound dressing will gain popularity within the pharmaceutical industry, among plastic surgeons and, not the least, among patients. In our opinion, approaches should focus on unsolved aspects, such as reducing the cost of wound care management and, more importantly, alleviating pain in patients resulting from wound dressings. Therefore, we can ask the following questions: are the dressings based on biopolymers and molecular biology less expensive and can they reduce pain, or should other approaches be outlined? Can the incorporation of bioactive components of plants with analgesic and/or anti-inflammatory properties into proposed smart wound dressings (designed as a three-module product) be an effective solution supported by significant scientific evidence? Another proposed approach is the use of cold atmospheric plasma for wound treatment, as demonstrated in a recent paper by our research group [186]. The cold atmospheric plasma itself consists of a highly reactive mixture of ions and electrons, reactive molecules, excited particles, electric fields and, to some extent, ultraviolet radiation. However, what if the cold plasma were supplemented in terms of the biopolymers involved; for example, in plasma delivery? The use of plasma and its components combined with biopolymers or other products in the treatment of various wounds can be considered a point to be explored further.

## Figures and Tables

**Table 1:** Summary of various current wound healing approaches.

Therapeutic Product	Advantages	Disadvantages	Refs.
Commercial dressings (e.g., Aquacel (carboxymethyl-cellulose), Kaltostat (ALG), Carboflex (ALG), Carbonet (cellulose) [17]	readily available and easy for clinicians to use, available in many shapes and sizes, comfortable can be used on any type of wound prevent bacterial contamination, may be used on infected wounds (alginates) waterproof and impermeable, highly absorbent (foams, alginates) or provide moderate absorption of exudate (hydrocolloids) encourage autolytic debridement (hydrocolloids, alginates), non-adherent (alginates)	some may adhere to the wound bed not effective for moist wound healing, may cause desiccation of the wound bed (alginates) must be changed frequently (often require a secondary dressing; e.g., foams, alginates), an aspect that increases the cost can leave a residue inclined to infection and cannot be used in the presence of infection (hydrocolloids) difficult to store and less choice/flexibility in indications for use (composites)	[8,18]
Biopolymer dressings	bioactive unique biochemical properties function in vivo and on the human body	poor mechanical features rapid biodegradability in vivo	[19,20]
PRP derivatives	economic, since they do not require complex equipment option to be allogeneic easy to obtain, manipulate and assemble with biomaterials possibility of reconstitution through rehydration immediate availability safe and effective (preclinical and a few clinic studies) favourable effects in regenerative medicine lack of immunological reaction risk, PRP being an autologous product minor adverse side effects	lack of regulatory requirements minor contamination and disease transmission possibilities if not allogeneic require optimization and standardization procedures lack of large clinical trials lack of consensus regarding PRP preparation techniques	[21,22]
Growth factors	regulate cellular responses required for wound healing more rapid skin regeneration	low in vivo stability restricted absorption elimination by exudation prior to reaching the wound area unwanted side effects increased costs may cause local allergic reactions	[23]

**Table 2:** Biopolymeric dressings/devices evaluated in clinical trials [45].

No.	Biopolymers Used for the Investigated Support	Conditions	Identifier/Status
1	Collagen/ type III collagen/ type I calf collagen matrix	Wounds	NCT02314416 Withdrawn 2015
2		Diabetic foot ulcers	NCT03010319 Completed 2021
3		Diabetic foot ulcers	NCT01270633 Terminated 2017 (business decision)
4		Diabetic foot ulcers	NCT01729286 Terminated
5		Localized gingival recessions	NCT02206009 Completed
6		Split-thickness skin grafts Wound healing	NCT03334656 Recruiting
7	Fibrin	Artificially induced wounds	NCT01253135 Completed 2015
8	Silk fibroin	Donor-site wounds	NCT01993030 Completed with results
9		Late complications from skin graft infection of skin donor site; pain	NCT02091076 Completed 2016
10	Silk sericin	Late complications from skin graft infection of skin donor site; pain	NCT01539980 Completed 2015
11	Silk sericin and collagen	Wound healing; wound surgery Donor-site complications	NCT04743375 Recruiting
12	Cellulose/ carboxymethyl cellulose/ hydroxyethyl cellulose	Venous ulcers	NCT00446823 Completed
13		Pemphigus Pemphigoid	NCT02365675 Unknown
14		Burns	NCT02318056 Withdrawn (reorganization)
15		Venous leg ulcers	NCT02921750 Completed 2020
16		Wound healing Patient comfort	NCT00428623 Completed 2007
17		Wound healing Total hip and total knee arthroplasty	NCT01258270 Completed 2013
18		Diabetic foot ulcers	NCT02667327 Terminated
19	Chitosan	Postpartum bleeding Vaginal lacerations Cervical lacerations	NCT01373801 Unknown as of 2011
20		Tooth extraction	NCT03108365 Completed
21		Cesarean wounds Scars; previous cesarean section	NCT04211597 Completed 2019
22	Poly-N-acetyl glucosamine	Venous stasis ulcers Venous insufficiency	Terminated; completed 2013
23	Alginate/ calcium alginate	Chronic wounds (>6 weeks)	NCT05009576 Completed 2021
24		Impaired wound healing Postoperative deep wound infections	NCT02198066 Completed 2014
25		Pilonidal sinuses	NCT02011802 Completed 2017
26	Hyaluronic acid	Wound healing	NCT03668665 Withdrawn (last update 2019)
27		Pilonidal cysts	NCT02485860 Recruiting
28		Free gingival grafts	NCT04390100 Completed 2020
29		Wound healing Complications	NCT02534415 Completed 2015
30	Oxidized regenerated cellulose and collagen	Venous ulcers Diabetic foot ulcers	NCT02845466 Unknown
31	Alginate and high-G cellulose	Pilonidal cysts/fistulas	Terminated (due to COVID-19 pandemic)
32	Carboxymethyl cellulose and sodium alginate	Diabetic foot ulcers Neuropathic diabetic foot ulcers	NCT03700580 Completed 2018

**Table 3:** PRP biopolymer-based products.

PRP Biopolymer-Based Products	Condition	Ref.
Polycaprolactone/gelatin filled with citicoline-bearing PRP gel	Autografting approach for peripheral nerve injury (PNI) treatment	[143]
Rhizochitosan and PRP	Full-thickness wound model	[144]
HA core-shell particles, loaded with PL and coated with calcium alginate, embedded in a VCM-containing alginate matrix	Chronic skin ulcers	[145]
Lyophilized Col sponge coated with PRP	Wounds Human periosteal fibroblasts Diabetic mice model	[146]
Freeze-dried platelet lysate encapsulated in Col, hASCs encapsulated in collagen plus platelet lysate beads	Wounds; in vitro tests: scratch wound assay, chick chorioallantoic membrane test	[147]
ALG/silk sericrin vs. freeze-dried platelet lysate/ALG/silk sericrin vs. alginate/freeze-dried platelet lysate (FD-PL)	Full-thickness chronic wounds in mouse model C57/BL6; granulation tissue, early inflammation, collagen deposition, fibroblast maturation, re-epithelialization, neovascularization	[148]
C-hPL, CL-hPL and L-hPL groups plus gelatin Human platelet lysate C-hPL: the cryopreservation hPL group CL-hPL: cryopreservated and lyophilized; L-lyophilized	Full-thickness wounds in male C57bl6J/Jcl mice Histology in mice: wound area, neovascularization, granulation tissue formation	[149]
Gelatin hydrogel (GH) sheet impregnated with PRP FD-PL vs. different concentrations of FD-PL	Full-thickness wounds in C57BL6J/Jcl mice; histology: H&E, Azan and anti-CD31	[150]
Cs/SF nanosilver loaded with FD-PRP (freeze-dried)	Wounds in BALBc mice; wound moisture retention and promotion of healing	[151]
FD-PRP plus carboxymethyl cellulose (CMC) (wafers) vs. FD-PRP powder	Wounds in a rat wound model	[152]
PRP plus a Col sponge (Collacote)	Apicomarginal defects: clinical study	[153]
Injectable hydrogel with a composite of chitosan, silk fibroin and PRP	Diabetic skin ulcer: in vitro and in vivo rat model	[154]
Carboxymethyl Cs gelatin microspheres loaded with gentamycin sulfate and PRP	Treatment of chronic and infected wounds in a Sprague–Dawley rats model	[155]
PRP and Cs dressing	*Candida albicans*-infected burn wound model in Wistar rats	[156]
Oxidized dextran/peptide-modified hyaluronic acid and PRP hydrogel	*E. coli*-, *S. aureus*- and *P. aeruginosa*-infected wounds in diabetic mouse model	[90]

**Table 4:** Growth factor biopolymer-based products.

Growth Factor Biopolymer-Based Products	Condition	Ref.
Film-forming spray of water-soluble chitosan containing hEGF-liposomes	Wound in male mice of the Swiss Webster strain	[180]
Cs-ulvan hydrogel incorporated in cellulose nanocrystal loaded with epidermal growth factor	Full-thickness skin wound in Balb/c mice	[181]
Cs/poloxamer-based thermosensitive hydrogels containing zinc gluconate/recombinant human epidermal growth factor	Scald wound model	[182]
Cs and EGF spray	Full-thickness wound in Wistar rat	[183]
Injectable hydrogel with sodium ALG, dextran, PDGF-BB and bone marrow-derived mesenchymal stem cells (BMSCs)	Full-thickness excisional wound model in C57BL/6 mice	[184]
Three dimensional porous collagen/chitosan scaffolds with selenium nanoparticles and fibroblast growth factor 2 (FGF2-STAB^®^)	In vitro and ex vivo evaluation	[185]
